# Supplementary material for: Mechanisms governing the pioneering and redistribution capabilities of the non-classical pioneer PU.1
Source: Nat Commun. 2020 Jan 21;11:402. doi: 10.1038/s41467-019-13960-2 (PMC6972792; doi:10.1038/s41467-019-13960-2)
Supplement: Supplementary file 7 — Source data [file 41467_2019_13960_MOESM7_ESM.zip › Source_Data/FigureS2/FigureS2B_MotifScanOutput/KmeansCluster12/homerResults/motif6.similar.html]

motif6

## Information for motif6

A
C
T
G
C
G
A
T
A
G
C
T
A
T
G
C
A
G
T
C
G
A
C
T
A
G
T
C
G
A
C
T
A
G
C
T
A
G
C
T
C
G
A
T
A
T
C
G
  
Reverse Opposite:  

T
A
G
C
G
C
T
A
T
C
G
A
T
C
G
A
C
T
G
A
T
C
A
G
C
T
G
A
C
T
A
G
T
A
C
G
T
C
G
A
G
C
T
A
T
G
A
C
  

|  |  |
| --- | --- |
| p-value: | 1e-42 |
| log p-value: | -9.732e+01 |
| Information Content per bp: | 1.708 |
| Number of Target Sequences with motif | 113.0 |
| Percentage of Target Sequences with motif | 6.29% |
| Number of Background Sequences with motif | 531.8 |
| Percentage of Background Sequences with motif | 1.16% |
| Average Position of motif in Targets | 103.2 +/- 55.2bp |
| Average Position of motif in Background | 93.6 +/- 70.1bp |
| Strand Bias (log2 ratio + to - strand density) | 0.0 |
| Multiplicity (# of sites on avg that occur together) | 1.00 |
| Motif File: | file (matrix) reverse opposite |

### Similar de novo motifs found

|  |  |  |  |  |  |  |  |
| --- | --- | --- | --- | --- | --- | --- | --- |
| Rank | Match Score | Redundant Motif | P-value | log P-value | % of Targets | % of Background | Motif file |
| 1 | 0.798 | C G T A C T G A C G T A C G T A C T A G C G T A C T A G T C A G C T G A T G C A T A C G C G A T A C T G | 1e-38 | -87.974768 | 3.51% | 0.32% | motif file (matrix) |
